# Increased S1P expression in osteoclasts enhances bone formation in an animal model of Paget's disease

**DOI:** 10.1002/jcb.29861

**Published:** 2020-10-27

**Authors:** Yuki Nagata, Kazuaki Miyagawa, Yasuhisa Ohata, Daniela N. Petrusca, Gabriel M. Pagnotti, Khalid S. Mohammad, Theresa A. Guise, Jolene J. Windle, G. David Roodman, Noriyoshi Kurihara

**Affiliations:** ^1^ Department of Medicine/Hematology‐Oncology Indiana University Indianapolis Indiana USA; ^2^ Department of Medicine/Endocrinology Indiana University Indianapolis Indiana USA; ^3^ Department of Human and Molecular Genetics Virginia Commonwealth University Richmond Virginia USA; ^4^ Roudebush VA Medical Center Indianapolis Indiana USA

**Keywords:** bone formation, osteoclasts, Paget's bone disease, S1P, S1PR3, SphK‐1

## Abstract

Paget's disease (PD) is characterized by increased numbers of abnormal osteoclasts (OCLs) that drive exuberant bone formation, but the mechanisms responsible for the increased bone formation remain unclear. We previously reported that OCLs from 70% of PD patients express measles virus nucleocapsid protein (*MVNP*), and that transgenic mice with targeted expression of *MVNP* in OCLs (*MVNP* mice) develop bone lesions and abnormal OCLs characteristic of PD. In this report, we examined if OCL‐derived sphingosine‐1‐phosphate (S1P) contributed to the abnormal bone formation in PD, since OCL‐derived S1P can act as a coupling factor to increase normal bone formation via binding S1P‐receptor‐3 (S1PR3) on osteoblasts (OBs). We report that OCLs from *MVNP* mice and PD patients expressed high levels of sphingosine kinase‐1 (SphK‐1) compared with wild‐type (WT) mouse and normal donor OCLs. SphK‐1 production by *MVNP*‐OCLs was interleukin‐6 (IL‐6)‐dependent since OCLs from *MVNP*/*IL‐6* ^−/−^ mice expressed lower levels of SphK‐1. Immunohistochemistry of bone biopsies from a normal donor, a PD patient, WT and *MVNP* mice confirmed increased expression levels of SphK‐1 in OCLs and S1PR3 in OBs of the PD patient and *MVNP* mice compared with normal donor and WT mice. Further, *MVNP*‐OCLs cocultured with OBs from *MVNP* or WT mice increased OB‐S1PR3 expression and enhanced expression of OB differentiation markers in *MVNP*‐OBs precursors compared with WT‐OBs, which was mediated by IL‐6 and insulin‐like growth factor 1 secreted by *MVNP*‐OCLs. Finally, the addition of an S1PR3 antagonist (VPC23019) to WT or *MVNP*‐OBs treated with WT and *MVNP*‐OCL‐conditioned media (CM) blocked enhanced OB differentiation of *MVNP*‐OBs treated with *MVNP*‐OCL‐CM. In contrast, the addition of the SIPR3 agonist, VPC24191, to the cultures enhanced osterix and Col‐1A expression in *MVNP*‐OBs treated with *MVNP‐*OCL‐CM compared with WT‐OBs treated with WT‐OCL‐CM. These results suggest that IL‐6 produced by PD‐OCLs increases S1P in OCLs and S1PR3 on OBs, to increase bone formation in PD.

## Graphical abstract

Model for the effects of sphingosine kinase‐1/sphingosine‐1‐phosphate/S1P‐receptor‐3 (SphK‐1/S1P/S1PR3) in abnormal bone remodeling in Paget's disease (PD). Measles virus nucleocapsid protein (MVNP) in PD‐osteoclasts (OCLs) induces interleukin‐6 (IL‐6), which upregulates insulin‐like growth factor 1 (IGF‐1) and Sphk1. SphK‐1 enhances S1P levels in OCLs, and IL‐6 and IGF‐1 increase S1PR3 on osteoblast (OBs). S1P then increases OB differentiation and bone formation via enhanced S1PR3 expression on OBs.

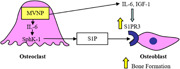

## 1 INTRODUCTION

Paget's disease (PD) is characterized by increased numbers of abnormal osteoclasts (OCLs) that drive exuberant bone formation, but the mechanisms responsible for the increased bone formation remain unclear.^1^, ^2^, ^3^ Both environmental and genetic factors have been implicated in the etiology of PD.^4^ We previously reported that OCLs from the majority of PD patients we studied express measles virus nucleocapsid protein (*MVNP*), and that transgenic mice with targeted expression of *MVNP* in OCLs (*MVNP* mice) develop bone lesions and abnormal OCLs characteristic of PD. MVNP expression in OCLs from PD patients or *MVNP* mice induced high levels of interleukin‐6 (IL‐6) in OCLs,^5^, ^6^ which was required for *MVNP* mice to develop pagetic‐like bone lesions that demonstrated highly localized increased bone resorption and formation.^7^, ^8^ Although these results suggest that the high IL‐6 levels in OCLs expressing *MVNP* increased local bone formation, it was unclear if the effects of IL‐6 on bone formation in PD were direct or mediated indirectly via other factors. More recently, we found that the high levels of IL‐6 in OCLs from PD patients and *MVNP* mice induced increased levels of insulin‐like growth factor 1 (IGF‐1) and ephrinB2 in OCLs and increased EphB4 expression on osteoblasts (OBs).^9^ IGF‐1 enhanced OB differentiation in *MVNP* mice.^9^ However, it is unknown if additional OCL‐derived factors also play a role in the exuberant bone formation characteristic of PD.

Sphingosine‐1‐phosphate (S1P) has fundamental functions in development, immunity, and as recently recognized, bone metabolism.^10^, ^11^, ^12^, ^13^, ^14^, ^15^, ^16^, ^17^ Extracellular S1P signals through G protein‐coupled receptors, S1PR1–S1PR5.^15^ S1P is synthesized by ceramide deacylation followed by phosphorylation by sphingosine kinase 1 (SphK‐1) and SphK‐2. Its concentrations in vivo are tightly regulated by dephosphorylation by two S1P‐specific phosphatases and three lipid phosphate phosphatases. S1P also undergoes irreversible degradation to phosphoethanolamine and 2‐hexadecanal by a single enzyme, S1P lyase.^12^ S1P's effects on bone homeostasis have been reported to be mainly through its effects on bone remodeling by regulating the migration of OCL progenitors in the circulation to the bone.^10^, ^11^, ^16^ Ishii et al.^18^, ^19^ showed that an S1P gradient between bone and plasma regulates OCL precursor migration. S1P in the plasma attracts OCL progenitors away from the bone by binding S1PR1 on OCL precursors, while S1P binding to S1PR2 on OCL precursors inhibits OCL precursor migration from the bone.

Numerous studies reported that S1P could affect OB behavior. OCL‐secreted S1P stimulates receptor activator of NF‐κB‐ligand (RANKL) production in OBs and promotes OB differentiation.^20^ Further, cathepsin K deletion in OCLs stimulates SphK‐1 activity that increases S1P expression and bone formation,^21^ via S1P binding to S1PR3 on OBs.^10^ These results suggest that S1P plays an important role in regulating OCL and OB activity. However, the role of S1P in PD is unclear. Therefore, in this report, we investigated the role and regulation of S1P and S1PRs in OCL and OB differentiation in PD, using OCLs and OBs from *MVNP* mice, and bone samples from a PD patient and a normal donor.

## 2 MATERIALS AND METHODS

### 2.1 Antibodies and reagents

Runx2 (sc‐10758), NFATc‐1 (sc‐7294, clone 7A6), EDG‐3 target for S1PR3 (sc‐30024, clone [H‐70]), and α‐tubulin (sc‐58666, clone4G1) antibodies were from Santa Cruz. Anti‐osterix antibody (ab22552) was purchased from Abcam Inc. Antibodies recognizing phospho‐Akt (Ser473) (#9271), Akt (#9272), phospho‐Erk1/2 (Thr202/Tyr204) (#9101), Erk1/2 (#9102), and glyceraldehyde 3‐phosphate dehydrogenase (#2118) on Western blots were purchased from Cell Signaling Technology. Anti‐phospho‐SphK1 (Ser225) antibody (LS‐C26925) was purchased from Life Span Bioscience. Anti‐SphK‐1 antibody (#ABN435) and anti‐collagen type1A (Col‐1A) (#AB765P) were obtained from Millipore. VPC 23019 (Cat# 4195) was purchased from TOCRIS Bioscience and VPC24191 (857365 P) was purchased from Sigma‐Aldrich.

### 2.2 Generation of MVNP, MVNP/IL‐6^−/−^, and IL‐6^−/− ^mice

Animal studies were approved by the Institutional Animal Care and Use Committees at Virginia Commonwealth University and Indiana University School of Medicine. Transgenic mice expressing *MVNP* under the control of the mouse *TRAP* promoter were generated as previously described.^8^ Global *IL‐6* ^−/−^ mice were generated by Kopf et al.^22^ and obtained from the Jackson Laboratory (stock number 002650). Mice were interbred with *MVNP* mice to generated *MVNP*/*IL‐6* ^−/−^ mice. All data are from mice generated from this *MVNP*/*IL‐6* ^−/−^ colony (not from the parental colonies).

### 2.3 Bone sections from a PD patient and a normal donor

Resin‐embedded bone sections from a PD patient and a healthy donor were generously provided by Dr. David D. Dempster (Department of Clinical Pathology and Cell Biology, Columbia University, New York, NY, USA). A transiliac crest bone biopsy was taken from a 58‐year‐old female patient with PD and a 30‐year‐old female healthy control who were treated with calcein and tetracycline before sampling. The biopsy sample was embedded without decalcification in methyl methacrylate. The bone sections were then processed as previously described by Gomes et al.^23^ SphK‐1 was detected with an anti‐SphK‐1 antibody (Life Span Bioscience) and S1PR3 was detected with an anti‐S1PR3 antibody (Santa Cruz). Briefly, bone sections were deacylated in a 1:1 mixture of xylene and chloroform, rehydrated in graded alcohol solutions, and then decalcified with 1% acetic acid. After rinsing with distilled water, and treatment with 0.1% Tween 20 in phosphate‐buffered saline, endogenous peroxidase activity was inhibited by a mixture of 3% hydrogen peroxide in methanol for 30 min, followed by two water washes. The samples were incubated with avidin‐biotin solutions and with 5% normal serum from the same species of secondary antibody with 1% bovine serum albumin to block nonspecific bindings. Histologic evaluation was performed under bright‐field microscopy. The detailed histological examinations of bone sections from this PD patient and normal donor were previously reported.^4^ Human studies were approved by the Indiana University Institutional Review Board.

### 2.4 Immunohistochemistry analyses

Femurs from male and female WT or *MVNP* mice (12 months of age) were fixed in 10% buffered formalin and completely decalcified in 10% ethylenediaminetetraacetic acid (EDTA) at 4°C for 20 days and embedded in paraffin. Five‐micrometer longitudinal sections were cut and mounted on glass slides. Deparaffinized sections were treated with 1% horse serum for one hour, then primary antibodies for SphK‐1 and S1P3 or control immunoglobulin G (IgG) (Cell Signaling) were added and the slides incubated overnight. The sections were then stained with anti‐rabbit IgG conjugated to peroxidase (Vector Laboratories). The representative image was semiquantitated using ImageJ. (https://imagej.net/Colour_Deconvolution). Preliminary experiments showed that SphK‐1 expression by OCLs or S1PR3 expression by OBs in bone sections from male versus female WT mice or male versus female *MVNP* mice did not differ significantly (Table S1). Therefore, marrow cells or bones from WT male and female or male and female *MVNP* mice were combined in the experiments below to minimize the number of mice used.

### 2.5 Preparation of bone lysates for protein analysis

Tibias and femurs were dissected from 12‐month‐old male and female WT and *MVNP* mice, frozen immediately in liquid nitrogen, and reduced to powder using a small mortar and pestle. Total proteins were extracted from WT or *MVNP*‐derived bones in lysis buffer overnight at 4°C, the male or female WT or MVNP lysates combined and 25 μg of protein/lane subjected to Western blot analysis using appropriate antibodies.

### 2.6 Formation of mature OCLs in mouse bone marrow cultures

Bone marrow cells flushed from long bones of WT, *MNVP*, *MVNP/IL‐6* ^−/−^, or *IL‐6* ^−/−^ mice were cultured with 10% fetal calf serum (FCS) in α‐minimum essential medium (αMEM) in 10‐cm dishes (2.5 × 10^7^ cells/dish) for overnight, then nonadherent marrow cells from these mice were harvested and enriched for CD11b+ mononuclear cells using the Miltenyi Biotec MACS (Magnetic Cell Sorting) system. These cells were incubated with hM‐CSF (10 ng/ml) (R&D System) for 3 days (CFU‐GM), then the cells treated with hRANKL (50 ng/ml) for 4 days as previously described.^5^, ^24^ At the end of cultures, trypsin‐EDTA (Corning) was added and cultures incubated for 3 min to remove nonosteoclastic cells and thereby enrich the concentration of OCLs in the cultures. The cultures were stained for TRACP, and TRACP‐positive multinucleated cells (≥3 nuclei/cell) were scored as OCLs.

### 2.7 Isolation and culture of primary OB‐precursors

After flushing the bone marrow from tibias of WT or *MVNP* mice, tibias were cultured in αMEM with 10% FCS for 7–10 days. The bones were then placed in 60‐mm dishes, and the cultures continued in αMEM containing 10% FCS until cells growing out of the bones formed a confluent monolayer. The original bones were removed and the out‐growth cells from the bones were treated with 0.25% trypsin and 0.05% EDTA for 10 min at 37°C. These cells were used as primary OBs without further passage. The primary OBs (2 × 10^5^ cells/well in six‐well plate) were cultured in αMEM containing 10% FCS for 7–14 days.^25^ The cell lysates were collected and analyzed for protein expression.

### 2.8 Coculture of OCLs and OBs from WT and MVNP mice

OCLs (2.0 × 10^4^/well) derived from marrow cultures of WT or *MVNP*‐mice (as described in Section 2.6) were scraped with a rubber policeman, replated, and cultured with 50 ng/ml RANKL overnight. OBs (1.0 × 10^5^/well) were plated on top of the OCLs the next day, and the cells cocultured for 72 h.

### 2.9 Treatment of primary OB precursors with OCL‐CM in the presence or absence of a S1PR3 antagonist or agonist

Primary OB precursors (1 × 10^5^/well) derived from WT‐ or *MVNP*‐mice bones as described above were cultured in αMEM with 10% FCS containing 30% (v/v) of OCL‐CM (conditioned for 48 h by OCLs from WT or *MVNP* mice) or with vehicle (αMEM) for 48 h. Then, an S1PR3 antagonist (10 µM) or agonist (10 µM) was added and the OB precursors cultured for 72 h.

### 2.10 Immunoblotting of OCL lysates from WT, MVNP, MVNP/IL‐6^−/−^, and IL‐6^−/− ^mice

Total proteins were extracted from OCLs formed from OCL precursors derived from these genotypes, and the cell lysates (25 µg/lane) loaded on sodium dodecyl sulfate (SDS) gels using the Bio‐Rad Mini‐gel System (Bio‐Rad Laboratories). The resolved proteins were transferred onto nitrocellulose membranes (TGX‐Membrane; Bio‐Rad) using Trans‐Blot Turbo System Bio‐Rad) and incubated in blocking solution (5% nonfat dry milk in TBST) for 1 h. Membranes were then exposed to primary antibodies overnight at 4°C and incubated with IgG horseradish peroxidase‐conjugated antibody for 1 h. The blots were washed and visualized by an Immobilon Western Chemiluminescent detection system (Thermo Fisher Scientific). Protein expression levels were quantitated by densitometry using ImageJ software (NIH).

### 2.11 The measurement of phosphorylation of SphK‐1 in OCLs

Preformed OCLs from WT or *MVNP* mice were cultured in αMEM + 10% FCS for 3 days. Cells were then starved by culturing in αMEM + 2% FCS for 24 h. Cells were then exposed to RANKL (50 ng/ml) for the indicated times and then lysed, fractionated by SDS‐polyacrylamide gel electrophoresis, and analyzed by immunoblot using antibodies recognizing phosphorylated Sphk‐1 and total SphK‐1.

### 2.12 S1P enzyme‐linked immunosorbent assay (ELISA) assays

OCLs (2.0 × 10^4^/ml) from WT and *MVNP* mice were isolated as described above and cultured with RANKL for 4 days. Conditioned media (CM) from these cultures were harvested at the end of the culture period, and the concentration of S1P was determined using an ELISA kit for mouse/human S1P (Echelon Biosciences, Inc.) according to the manufacturer's instructions.^26^ The assay was calibrated based on a standard curve using the S1P protein that was included in the ELISA kit. The results were determined by subtracting the S1P level of the culture media containing serum without cultured cells.

### 2.13 Statistical analysis

Significance was evaluated using a two‐tailed Welch's *t* test. When more than two treatment groups were compared, a one‐way analysis of variance with Tukey's test for repeated measures was used. Differences with *p* < .05 were considered significant.

## 3 RESULTS

### 3.1 SphK‐1 expression in OCLs from MVNPmice and a patient with PD disease

**Figure 1:**
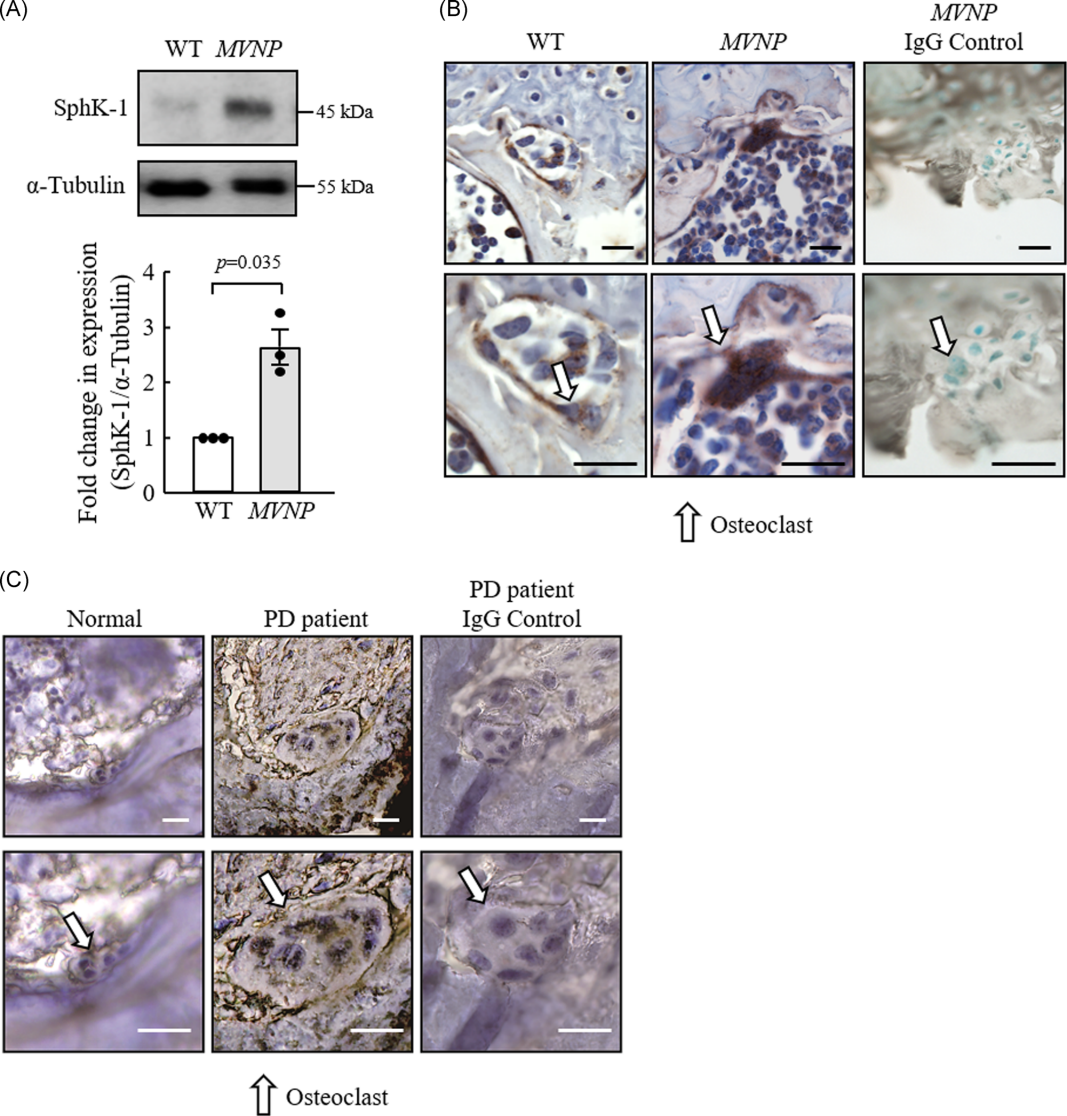
SphK‐1 expression is increased in bone lysates and OCLs from *MVNP* mice and a patient with Paget′s disease. (A) SphK‐1 expression in bones from 12‐month‐old of WT or *MVNP* mice. The bone extracts from the femur and tibia of one female and one male WT or *MVNP* mouse were combined and loaded on SDS gels, and SphK‐1 expression analyzed by Western blot analysis using an anti‐SphK‐1 antibody. α‐Tubulin was used as the loading control. The relative expression levels of SphK‐1 expressed compared with α‐tubulin with the basal ratio of SphK‐1/α‐tubulin for WT mice set at 1.0. Results are the mean ± *SEM* from three biological replicates. (B) SphK‐1 expression in OCLs in the distal femur from 12 months old WT and *MVNP* mice. Decalcified sections from femurs were stained with anti‐SphK‐1 antibody. The upper panels show low power views and the lower panels show high power views. The arrow indicates the OCLs. Immunostaining for anti‐SphK‐1 is brown. All scale bars represent 10 µm. (C) SphK‐1 expression in OCLs of transiliac crest bone sections from a normal donor and a patient with Paget′s disease. The upper panels show low power views and the lower panels show high power views. The arrow indicates the OCLs. Immunostaining for anti‐SphK‐1 is brown. All scale bars represent 10 µm. MVNP, measles virus nucleocapsid protein; OCLs, osteoclasts; PD, Paget′s disease; SphK‐1, sphingosine kinase‐1; SDS, sodium dodecyl sulfate; *SEM*, standard error of the mean; WT, wild type

Twelve months old mice were used for these studies because OCLs from *MVNP* mice at that age and older consistently express a Pagetic phenotype in vitro.^5^, ^6^, ^7^, ^8^, ^9^ Therefore, we determined SphK‐1 expression levels in bone extracts (tibia and femur) from 12 months old WT and *MVNP* mice. SphK‐1 was detected in both WT and *MVNP* mice, and was increased 2.6‐fold in *MVNP* mice compared with WT (Figure 1A). We next determined SphK‐1 expression in distal femur sections from 12 months old WT and *MVNP* mice by immunohistochemistry. We found that SphK‐1 expression was increased in OCLs from *MVNP* mice compared with WT mice (Figure 1B). We semiquantified the ShpK‐1 staining in randomly selected 20 OCLs in three sections from the individual WT and *MVNP* mice. The OCLs from *MVNP* mice displayed a 2.4‐fold higher staining level than WT (*p* < .01 two‐tailed Welch's *t* test). We next examined SphK‐1 expression in transiliac crest bone sections from a normal donor and a PD patient by immunohistochemistry. We found that SphK‐1 expression was increased in OCLs from the PD patient compared with the normal donor (Figure 1C).

### 3.2 SphK‐1 expression is increased in OCLs from MVNP mice

**Figure 2:**
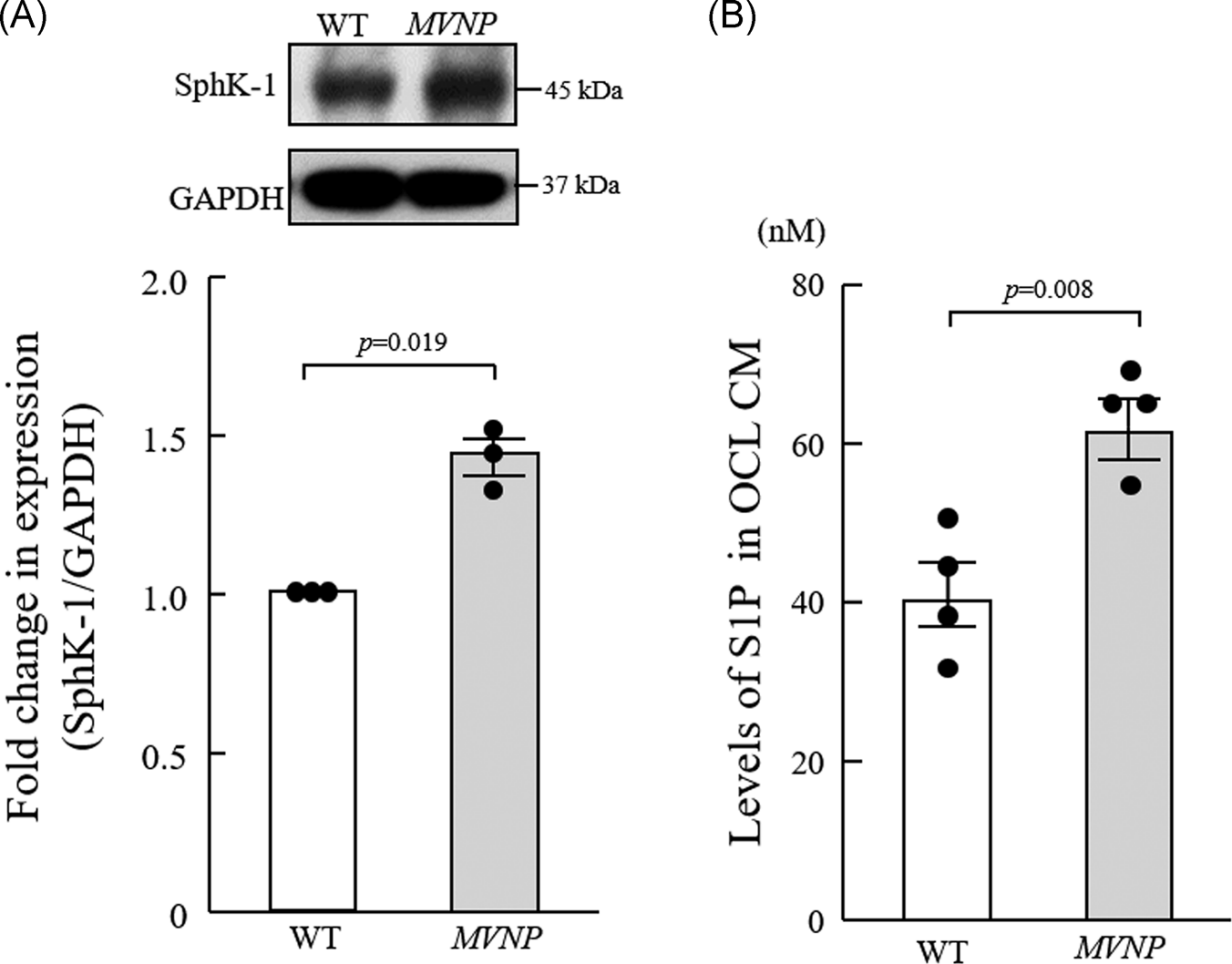
Expression levels of SphK‐1 and S1P in OCLs derived from WT‐ or *MVNP*‐mice bone marrow cultures. (A) SphK‐1 expression in lysates of OCLs derived from cocultures of equal numbers of male and female marrow cells from one male and one female WT or one male or female *MVNP* mouse was determined by Western blot analysis as described in Materials and Methods. The basal ratios of SphK‐1/GAPDH levels for OCLs from WT mice were set at 1.0. Results are the mean ± *SEM* from three biological replicates. The data were analyzed using a two‐tailed Welch′s *t* test. (B) S1P levels in media conditioned for 3 days by purified OCLs (2.0 × 10^4^/ml) from cultures of marrow cells from 12 months old mice as described above. S1P levels were determined as described in Materials and Methods. The results are shown as the mean ± *SEM* for four biological replicates. The data were analyzed using a two‐tailed Welch′s *t* test. ELISA, enzyme‐linked immunoabsorbent assay; GAPDH, glyceraldehyde 3‐phosphate dehydrogenase; MVNP, measles virus nucleocapsid protein; OCLs, osteoclasts; SDS, sodium dodecyl sulfate; *SEM*, standard error of the mean; S1P, sphingosine‐1‐phosphate; SphK‐1, sphingosine kinase‐1; WT, wild type

We then determined SphK‐1 expression levels in purified OCLs derived from *MVNP* or WT mouse bone marrow cultures. SphK‐1 expression was increased 1.5‐fold in OCLs from *MVNP* mice compared with WT mice (Figure 2A). Further, S1P levels in media conditioned by purified OCLs for 3 days showed that OCLs from *MVNP* mice released 1.5‐fold more S1P compared with WT OCLs (Figure 2B).

### 3.3 IL‐6 increases SphK‐1 expression in OCLs from MVNP mice

**Figure 3:**
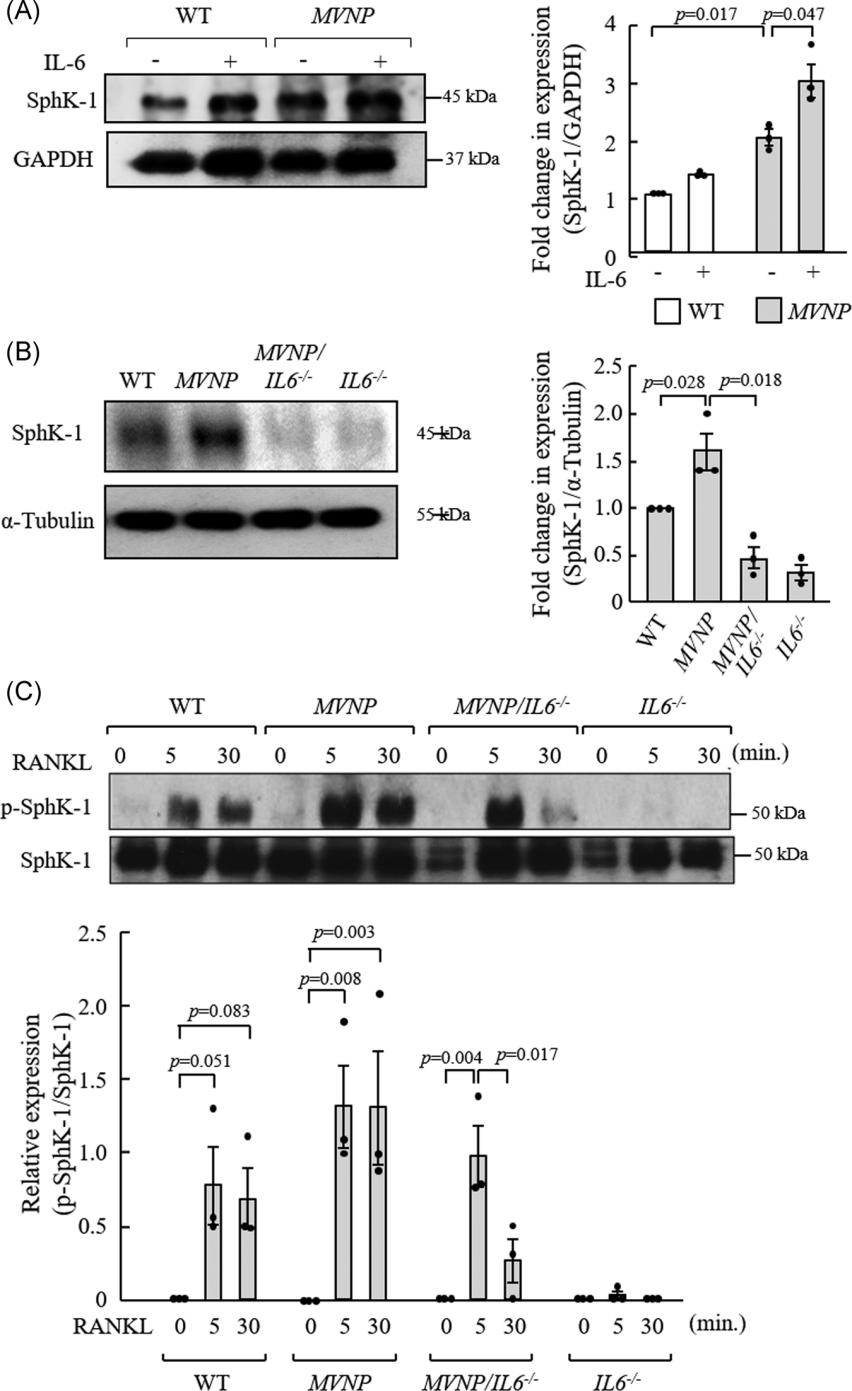
IL‐6 increases SphK‐1 expression in OCLs of WT and *MVNP* mice. (A) Effects of IL‐6 treatment on SphK‐1 expression in mature OCLs. Preformed OCLs derived from cocultures of CD11b (+) bone marrow cells from one female and male WT or *MVNP* mouse were cultured with/without 10 ng/ml of IL‐6 for 48 h. The cell lysates were then loaded onto SDS gels, and SphK‐1 expression analyzed as described in Materials and Methods. Results are the mean ± *SEM* from three biological replicates. The data were analyzed using ANOVA with Tukey's test. (B) SphK‐1 expression in bone extracts from 12 months old WT, *MVNP*, *MVNP*/*IL‐6* ^−/−^, and *IL‐6* ^−/−^ mice. Bone extracts derived from the femurs and tibia of one female and male mouse of each genotype were loaded onto SDS gels, and SphK‐1 expression analyzed as described in Materials and Methods. Results shown are representative of those from three biological replicates. The ratio of SphK1/α‐tubulin was evaluated by ImageJ. The basal ratio of SphK‐1/α‐tubulin in WT mice was set at 1.0. The data were analyzed using one‐way ANOVA with Tukey's test. (C) Effects of RANKL on SphK‐1 phosphorylation in OCLs from 12 months old WT, *MVNP*, *MVNP*/*IL‐6* ^−/−^, and *IL‐6* ^−/−^ mice. OCLs precursors from these mice were incubated with RANKL (50 ng/ml) for 5 or 30 min. and Phospho‐SphK‐1 was measured in cell lysates as described in Materials and Methods. These experiments were performed by combining lysates derived from one female and male mouse of each of the four genotypes. The results of the experiment shown were similar in three biological replicates. *p < .01 compared with 0 min treatment of OCLs precursors from each genotype. The data were analyzed using ANOVA with Tukey's test. ANOVA, analysis of variance; CD, cluster of differentiation; GAPDH, glyceraldehyde 3‐phosphate dehydrogenase; IL‐6, interleukin‐6; MVNP, measles virus nucleocapsid protein; OCLs, osteoclasts; *SEM*, standard error of the mean; SphK‐1, sphingosine kinase‐1; WT, wild type

OCLs from patients with PD express high levels of IL‐6,^24^ and MVNP expression in OCLs induces high IL‐6 expression levels that are essential for the formation of pagetic‐like OCLs and bone lesions in *MVNP*‐expressing mice.^4^ However, it is unknown if IL‐6 increases SphK‐1/S1P expression, which would in turn increase the S1P levels in *MVNP* mice. We found that IL‐6 treatment (10 ng/ml) of OCLs derived from CD11b+ OCL precursors from WT and *MVNP* mice increased SphK‐1 expression in WT OCLs and in *MVNP*‐expressing OCLs by 1.5‐fold compared with vehicle‐treated cultures (Figure 3A). Since the loss of IL‐6 prevents the development of pagetic OCLs and pagetic bone lesions in *MVNP* mice,^5^ we tested if IL‐6 regulates SphK‐1 expression in OCLs by measuring SphK‐1 expression in OCLs from WT, *MVNP*, *MVNP/IL‐6* ^−/−^, and *IL‐6* ^−/−^ mice. SphK‐1 expression in lysates of OCLs from *MVNP/IL‐6* ^−/−^ and *IL‐6* ^−/−^ mice was significantly decreased compared with WT and *MVNP*‐mouse lysates (Figure 3B). Loss of IL‐6 also decreased the expression of phospho‐SphK1 in RANKL‐treated OCLs derived from WT and *MVNP* mice (Figure 3C).

### 3.4 S1PR3 expression in OBs from WT and MVNPmice

**Figure 4:**
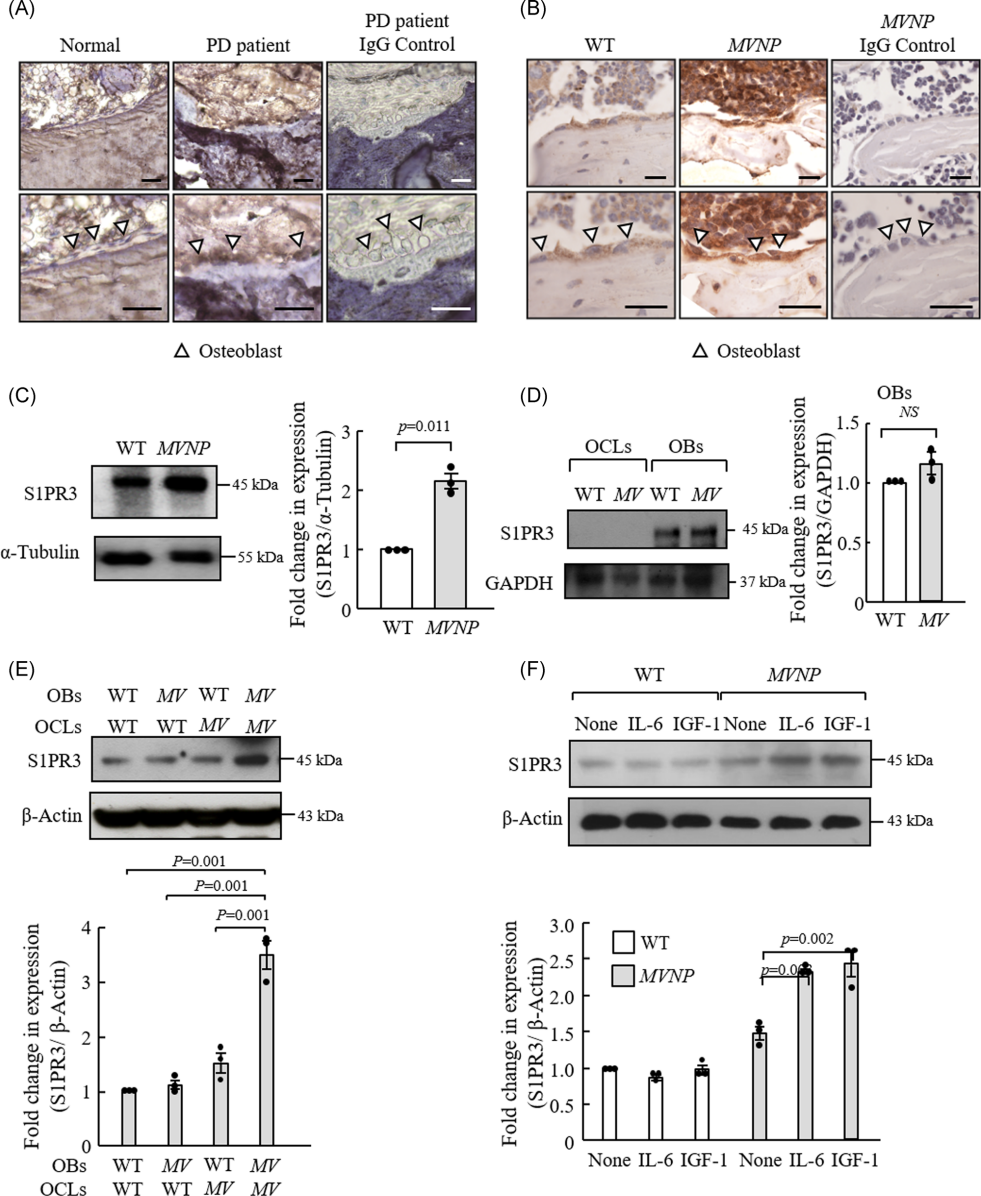
S1PR3 expression in OBs from *MVNP* mice and a patient with PD. (A) S1PR3 expression in OBs in transiliac crest bone sections from a normal donor and a PD patient. The upper panels show low power views and the lower panels show high power views. Immunostaining for S1PR3 is shown in brown. All scale bars represent 10 µm. (B) S1PR3 expression in OBs of vertebrae from WT and *MVNP* mice. Decalcified sections from the distal femur of 12 months old mice were stained with the anti‐S1PR3 antibody. The upper panels show low power views and the lower panels show high power views. Immunostaining for S1PR3 is shown in brown. All scale bars represent 10 µm. (C) S1PR3 expression in bone lysates from 12 months old WT or *MVNP* mice. The extracts from femurs of one female and one male mouse of each genotype were combined and loaded on SDS gels. S1PR3 expression was analyzed by Western blot analysis. The same membranes used in Figure 1A were reprobed and analyzed for S1PR3 expression. The basal ratio of S1PR3/α‐tubulin for WT mice was set at 1.0. Results are the mean ± *SEM* from three biological replicates. The data were analyzed using Welch′s *t* test. (D) S1PR3 expression in OCLs and OBs from 12 months old WT and *MVNP* mice. OCLs were isolated from cocultures of marrow from one male and one female WT or *MVNP*‐mouse. OBs were derived from outgrowth cells of bones from one male and female WT or *MVNP* mice. The OB lysates from male and female WT of *MVNP*‐bones were combined, and S1PR3 expression was analyzed by Western blots. The ratios of S1PR3/GAPDH were evaluated using ImageJ. (E) S1PR3 expression on OBs from cocultures of OCLs and OBs from 12 months old WT and *MVNP* mice. OBs derived from bones of one female and one male WT or *MVNP*‐mouse were combined for this assay. OCLs and OBs were purified and then cocultured for 3 days and the cell lysates from the OBs were assayed for S1PR3 expression by Western blot. The ratios of S1PR3/β‐actin were evaluated using ImageJ. The basal ratio of S1PR3/β‐actin for WT‐OBs/WT‐OCLs cocultures was set at 1.0. The data were analyzed using a one‐way ANOVA with Tukey's test. (F) Induction of S1PR3 expression by IL‐6 and IGF‐1 on OBs from 12 months old WT and *MVNP* mice. OBs derived from femurs from one female and male WT or *MVNP*‐mouse were combined and treated with IL‐6 (10 ng/ml) and/or IGF‐1 (10 ng/ml) for 72 h. S1PR3 expression was assayed by Western blot. The ratio of S1PR3/β‐actin was evaluated by ImageJ. The basal ratio of SphK‐1/β‐actin for vehicle treatment of WT‐OB culture was set at 1.0. The data were analyzed using a one‐way ANOVA with Tukey's test. ANOVA, analysis of variance; GAPDH, glyceraldehyde 3‐phosphate dehydrogenase; IGF‐1, insulin‐like growth factor 1; IL‐6, interleukin‐6; MVNP, measles virus nucleocapsid protein; OBs, osteoblasts; OCL, osteoclasts; PD, Paget's disease; S1PR3, sphingosine‐1‐phosphate receptor 3; *SEM*, standard error of the mean; WT, wild type

We then examined S1PR3 expression on OBs from WT and *MVNP* mice. We found that S1PR3 expression was increased in OBs from a PD patient compared with a normal donor (Figure 4A). Further, when we examined S1PR3 expression in OBs from WT and *MVNP* mice, the OBs in 12 months old *MVNP* mice were more strongly stained (Figure 4B). We semiquantified the S1PR3 staining in 30 randomly selected OBs from each section from three WT and *MVNP* mice. The OBs from *MVNP* mice had 2.5‐fold higher stain than WT (*p* < .01 two‐tailed Welch's *t* test). We then determined S1PRs expression levels in bone extracts (tibia and femur) from 12 months old WT and *MVNP* mice. S1PR3 expression was increased by twofold in bone extracts of *MVNP*‐mice compared with WT (Figure 4C). To determine if these results reflect S1P3 expression on OBs in the bone, we then examined the expression levels of S1PR3 in purified OBs and OCLs from WT and *MVNP* mice. OBs from WT and *MVNP* mice expressed similar levels of S1PR3, but S1PR3 expression in OCLs of WT and *MVNP* mice was very low compared with OBs (Figure 4D). Although S1PR3 in OBs was detectable in both WT and *MVNP* mice, only very low levels of S1PR1 and S1PR2 were found in OBs from both genotypes (data not shown). Because S1PR3 was increased in OBs in bone sections from a PD patient and MVNP mice, we then examined S1PR3 expression levels in cocultures of WT‐OCLs/WT‐OBs, WT‐OCLs/*MVNP*‐OBs, *MVNP*‐OCLs/WT‐OBs, and *MVNP*‐OCLs/MVNP‐OBs. As expected, cocultures of OCLs/OBs from *MVNP* mice induced the highest S1PR3 levels on OBs compared with other coculture combinations (Figure 4E).

We next determined if IL‐6 or IGF‐1 induced SIPR3 expression in the OCLs/OBs cocultures since IL‐6 and IGF‐1 are produced by *MVNP*‐OCLs.^12^ Western blot analysis of OBs from bone explants of WT and *MVNP* mice showed that S1PR3 expression was higher in OBs from *MVNP* mice and was further increased in *MVNP*‐OBs by either IL‐6 (10 ng/ml) or IGF‐1 (10 ng/ml). In contrast, IGF‐1 had no effect on S1PR3 expression by WT‐OBs (Figure 4F), consistent with the minimal increase in S1PR3 expression in cocultures of *MVNP*‐OCL with WT‐OBs.

### 3.5 S1P induced the expression of differentiation markers in OBs from WT and MVNP mice

**Figure 5:**
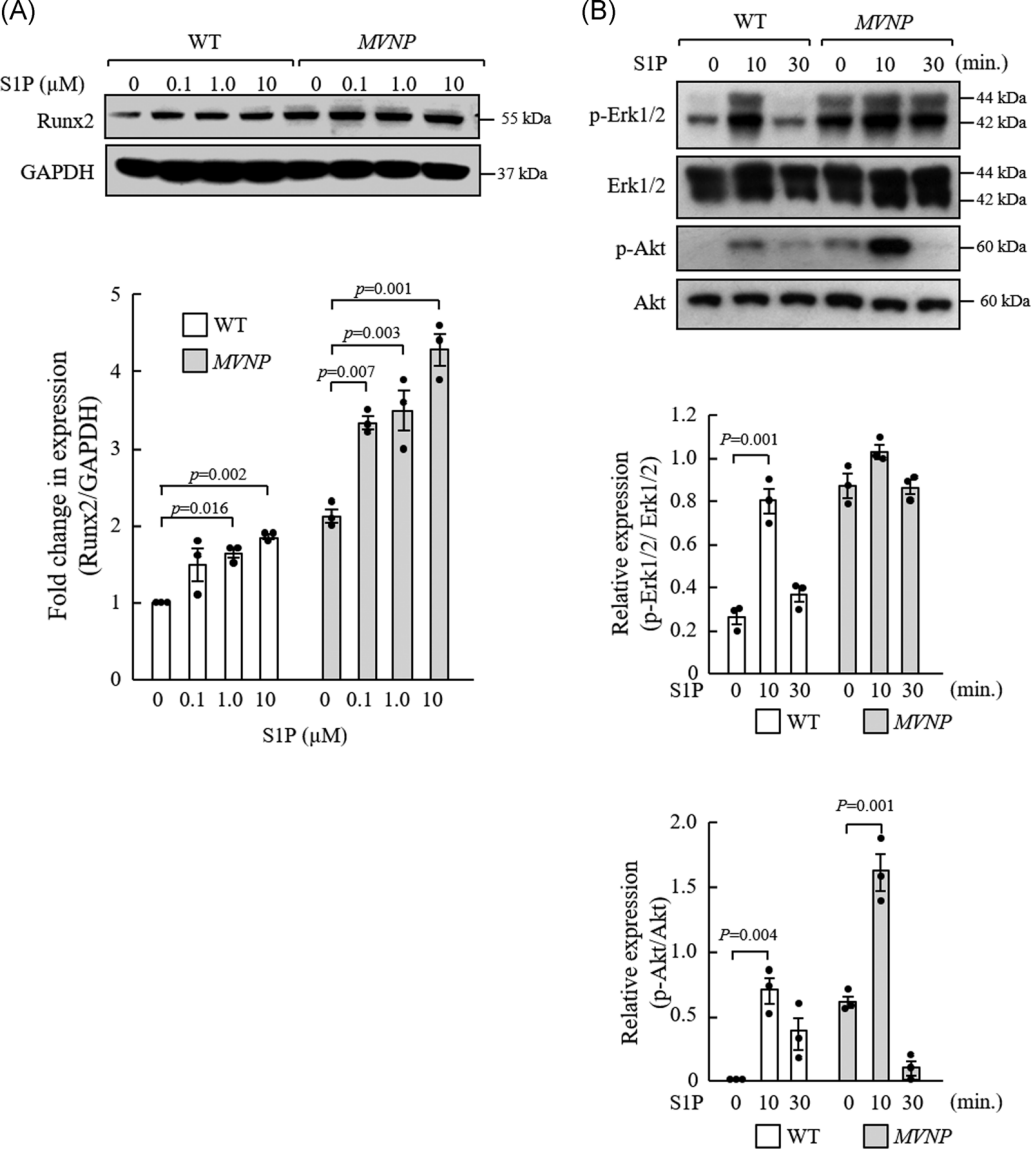
S1P increases OB differentiation of OBs from WT and *MVNP* mice. (A) Induction of Runx2 expression by S1P in OBs derived from long bones of 12 months old WT and *MVNP* mice. OBs from WT and *MVNP* mice were prepared as described in Figure 4 and were treated with S1P (0–10 µM) for 48 h. The cell lysates were collected and analyzed for Runx2 expression by Western blot as described in Materials and Methods. The basal ratio of Runx2/GAPDH for vehicle‐treatment of WT‐OBs culture was set at 1.0. The data were analyzed using one‐way ANOVA with Tukey's test. (B) Induction of Erk1/2 and Akt phosphorylation by S1P in OBs from 12 months old WT and *MVNP* mice derived as described in Figure 4. OBs were incubated with 10% FCS in αMEM overnight, then treated with S1P (10 µM) for the denoted time periods, and Erk 1/2 and Akt phosphorylation analyzed as described in Materials and Methods. The results of the experiment shown were similar in three biological replicates. ANOVA, analysis of variance; OB, osteoblast, FCS, fetal calf serum; GAPDH, glyceraldehyde 3‐phosphate dehydrogenase; MVNP, measles virus nucleocapsid protein; Runx2, runt‐related transcription factor 2; S1P, sphingosine‐1‐phosphate; WT, wild type

We then determined if S1P induces OB differentiation in WT and *MVNP* OBs by treating OB precursors from WT and *MVNP* mice with S1P for 3 days. S1P (10 µM) increased Runx2 expression in OBs from *MVNP* mice approximately twofold compared with controls without S1P treatment (Figure 5A). Since S1P enhanced Runx2 expression in OBs derived from WT and *MVNP* mice, we assessed if S1P activates MAPK and PI3K to induce OB differentiation and bone formation. As shown in Figure 5B, S1P activated Erk and Akt phosphorylation. Erk and Akt activation was detectable at 10 min in WT‐OBs treated with S1P and was already present at time 0 in OBs from *MVNP* mice. The increased Erk and Akt phosphorylation levels returned to basal levels at 30 min in OBs from WT and *MVNP* mice, although the p‐ERK1/2 levels were still elevated in *MVNP*‐OBs compared with WT‐OBs.

### 3.6 S1P induced by MVNP in OCLs enhances OB differentiation marker expression on OBs via S1PR3‐induced signaling

**Figure 6:**
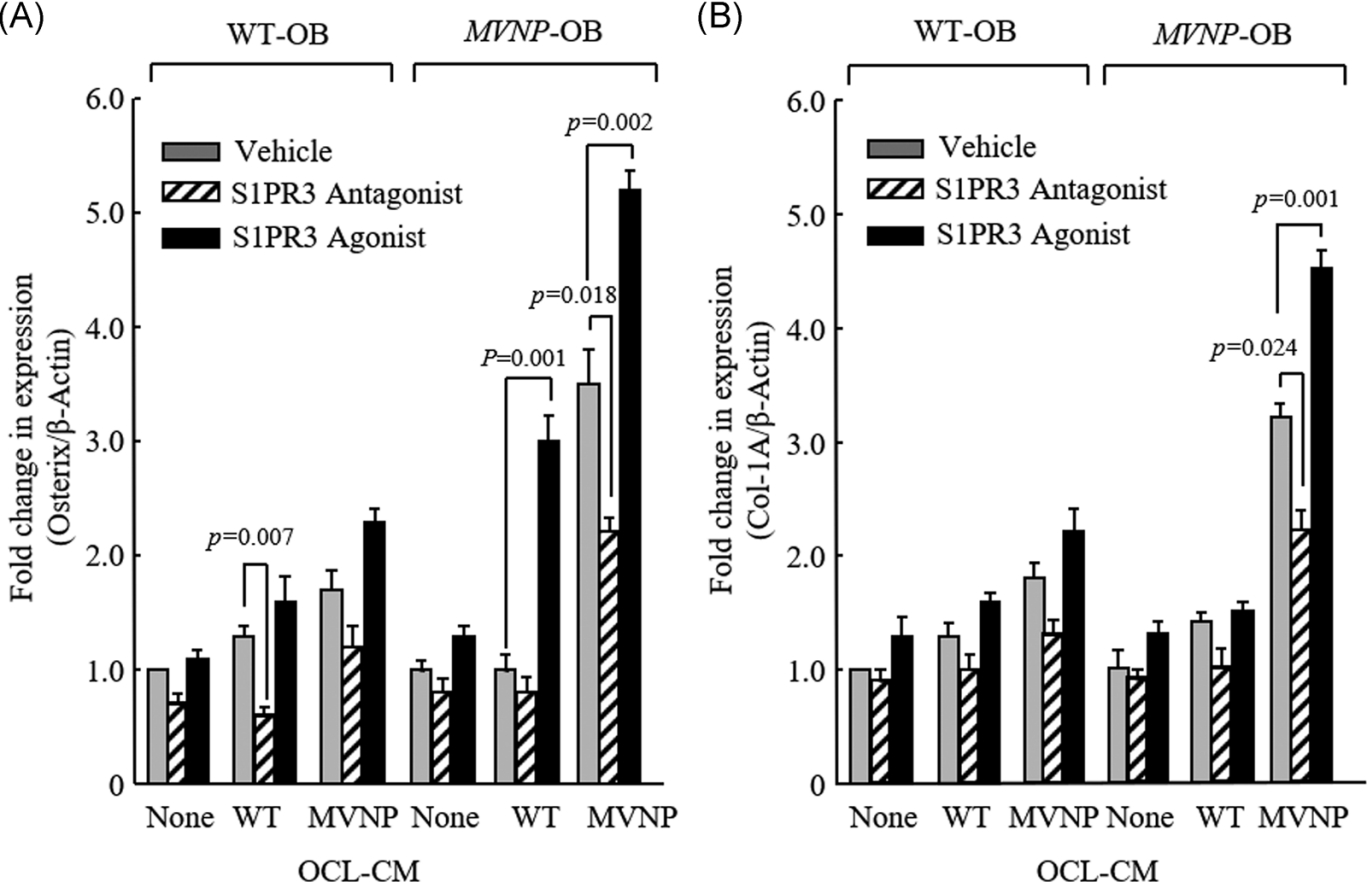
Effects of treatment with S1PR3 antagonist or agonist on osterix (A) and Col‐1A (B) expression induced by OCL‐conditioned media (CM). OBs from WT‐ or *MVNP*‐mouse bones from one male and one female WT or *MVNP* mouse were prepared and cultured for 3 days with OCL‐CM from *MVNP* or WT mice in the presence of either an S1PR3 antagonist (VPC23019)(10 µM) or an S1PR3 agonist (VPC24191) (10 µM), as described in Materials and Methods. The cell lysates from these cultures were then analyzed for expression of osterix and Col‐1A, as described in Materials and Methods. Expression levels of osterix and Col‐1A were compared with β‐actin by densitometry using ImageJ. The data are shown as mean ± *SEM* of four biological replicates. The data were analyzed using one‐way ANOVA with Tukey's test. For statistical analyses, results were compared with vehicle treatment of WT or *MVNP* OCL‐CM stimulated cultures. ANOVA, analysis of variance; Col‐1A, collagen type 1; MVNP, measles virus nucleocapsid protein; OBs, osteoblasts; OCL, osteoclasts; *SEM*, standard error of the mean; S1PR3, sphingosine‐1‐phosphate receptor 3; WT, wild type

To confirm that S1PR3 mediates the enhanced OB differentiation induced by S1P, we added an S1PR3 antagonist (VPC23019) (10 µM) or S1PR3 agonist (VPC24191) (10 µM) to cultures of OBs pretreated for 24 h with media conditioned by OCLs, from WT and *MVNP* mice and continued the MVNP and WT OB cultures in the CM for 3 days. We then measured in the OB lysates by Western blot of osterix, an OB transcription factor that is essential for OB differentiation and is downstream of Runx2, and Col‐1A, which is required for normal bone formation by OBs. VPC23019 decreased osterix levels in WT‐OBs treated with WT‐OCL‐CM and *MVNP*‐OB treated with *MVNP*‐OCL‐CM compared with vehicle treatment of the cultures (*p* < .05) (Figure 6A). In contrast, VPC24191 increased osterix levels in *MVNP*‐OB treated with WT‐OCL‐CM and *MVNP*‐OCL‐CM (*p* < .01) (Figure 6A). As expected, VPC24191 enhanced Col‐1A expression to a greater extent in *MVNP*‐OBs treated with *MVNP*‐OCL‐CM compare with vehicle treatment (*p* < .01) (Figure 6B).

## 4 DISCUSSION

We previously showed that *MVNP* expression in OCLs from *MVNP* mice and PD patients increases IL‐6 production, which upregulates expression of ephrinB2 and IGF‐1 in OCLs and EphB4 in OBs.^9^ These results suggest that enhanced coupling factor expression and increased IGF‐1 production by pagetic OCLs may contribute to the rapid bone formation that occurs in PD. To determine if additional OCL‐derived factors contributed to the increased bone formation in PD, we tested if S1P was also involved because OCL‐derived S1P was recently reported to increase bone formation by binding the S1PR3 on OBs.^10^

We found that SphK‐1 and S1P production was increased in *MVNP*‐expressing OCLs (Figures 1 and 2). Further, S1P production by *MVNP*‐OCLs was IL‐6‐dependent since SphK‐1 expression was markedly decreased in OCLs from *MVNP/IL‐6* ^−/−^ mice (Figure 3). Thus, S1P production was regulated by the autocrine/paracrine effects of OCL‐IL‐6, and decreasing OCL number and activity in PD should reduce S1P secretion in vivo. These results further support our previous findings that increased IL‐6 in PD‐OCLs plays a critical role in the enhanced OCL and OB activities in PD^9^ since IL‐6 produced by *MVNP*‐OCLs increased S1P production as well as expression of ephrinB2 and IGF‐1 production by OCL and EphB4 by OB.^9^

Additionally, immunohistochemical analysis of bones from a normal donor, a PD patient, WT and *MVNP* mice showed that OBs from the PD patient and *MVNP* mice expressed high levels of S1PR3 in vivo (Figure 4A,B), and that bone lysates from *MVNP* mice had increased expression of S1PR3 (Figure 4C). Our coculture studies of OCLs and OBs supported these in vivo findings and showed that OCLs from *MVNP* mice enhanced S1PR3 expression levels in OBs from *MVNP* but not WT mice (Figure 4E). As shown Figure 4D, S1PR3 levels in WT and MVNP mice OBs were similar, but S1PR3 expression was increased by treatment of *MVNP* but not WT OBs with IL‐6 or IGF‐1 (Figure 4F). These results suggest that OCL‐derived IGF‐1 induced by IL‐6 also increased S1PR3 in addition to their potential effects on OB differentiation via Erk and Akt pathways (Figure 5B). The inability of IL‐6 or IGF‐1 to increase S1PR3 in WT OBs suggests that additional factor(s) in vivo may prime OBs in *MVNP* mice to respond to IL‐6 and/or IGF‐1 to increase S1PR3 expression in PD‐OBs. The identity of this factor(s) is currently unknown.

Since increased S1PR3 mediated signaling via increased S1P and S1PR3 may contribute to the enhanced OB differentiation in *MVNP* mice, we determined if treating OBs from WT and *MVNP* mice with WT or MVNP‐OCL‐CM, which contained S1P (as shown in Figure 2B) in the presence of S1PR3 antagonist (VPC23019) or agonist (VPC24191) could block or enhance the effect of OCL‐CM on the expression of osterix and Col1A, factors required for bone formation. The S1PR3 agonist‐stimulated expression of these markers of OB differentiation in *MVNP*‐OB to a greater extent than WT‐OB treated with OCL‐CM from *MVNP* mice. In contrast, the S1PR3 antagonist inhibited the expression of these OB differentiation markers in WT‐OB to a greater extent than in *MVNP*‐OB (Figure 6). These results are consistent with our hypothesis that S1P increases new bone formation in PD patients expressing MVNP in OCLs, and that enhanced OB differentiation is due to in part to increased levels of S1PR3 on OB precursors. A recent study by Keller et al.^11^ supports our results that S1P from OCLs can regulate bone formation. They showed that calcitonin negatively regulates bone formation by inhibiting the release of the anabolic bone factor, S1P, from OCLs.

**Figure 7:**
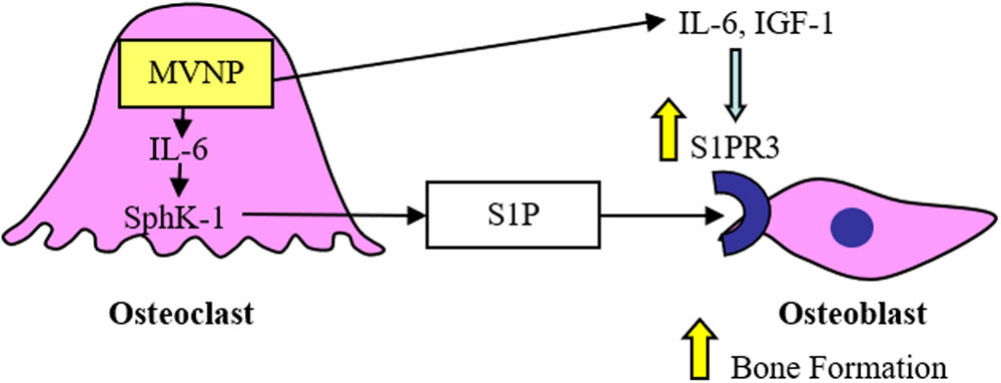
Model for the effects of SphK‐1/S1P/S1PR3 on the abnormal bone remodeling in PD. MVNP in PD‐OCLs induces IL‐6, which upregulates IGF‐1 and SphK‐1. SphK‐1 enhances S1P levels in OCLs and IL‐6 and IGF‐1 increase S1PR3 on OBs. S1P then increases OB differentiation and bone formation via enhanced S1PR3 expression on OBs. IGF‐1, insulin‐like growth factor 1; IL‐6, interleukin‐6; MVNP, measles virus nucleocapsid protein; OBs, osteoblasts; OCL, osteoclast; PD, Paget′s disease; S1P, sphingosine‐1‐phosphate; S1PR3, sphingosine‐1‐phosphate receptor 3; SphK‐1, sphingosine kinase‐1

Our current model for the potential action of S1P in PD is depicted in Figure 7. IL‐6 produced by *MVNP*‐expressing OCLs increases S1P production by OCLs as well as in combination with IGF‐1 promotes S1PR3 expression on OBs to increase bone formation. In this model, IL‐6 increases ephrinB2, IGF‐1, and SphK‐1/S1P in *MVNP* mice. These results suggest that OCL‐IL‐6 contributes to bone formation in PD by inducing OCL‐IGF1, which enhances bone formation via the upregulation of ephrinB2/EphB4 in OCLs and OBs, respectively,^9^, ^27^ and increases Shpk1/S1P/S1PR3 in OCLs and OBs in *MVNP* mice. These results further suggest that antagonists of S1PR3 may be useful to control the increased bone formation in patients with PD, who are unable to receive bisphosphonates. Our results also support recent reports by Weske et al.,^28^ who suggested that S1P‐based drugs could be a promising anabolic treatment for bone loss.

## CONFLICT OF INTERESTS

The authors declare that there are no conflict of interests.

## AUTHOR CONTRIBUTIONS

*Designed the study, interpreted the data, and wrote the manuscript*: G. David Roodman and Noriyoshi Kurihara. *Performed the experiments*: Yuki Nagata, Yasuhisa Ohata, Kazuaki Miyagawa, Daniela N. Petrusca, and Noriyoshi Kurihara. *Generated the transgenic mice and helped write the manuscript*: Jolene J. Windle. *Performed histological studies and analyses*: Gabriel M. Pagnotti, Khalid S. Mohammad, and Theresa A. Guise. All authors approved the submission of the manuscript.

## Supporting information

Supporting information.
