# Supplementary material for: Increased S1P expression in osteoclasts enhances bone formation in an animal model of Paget's disease
Source: J Cell Biochem. 2020 Oct 27;122(3-4):335–48. doi: 10.1002/jcb.29861 (PMC7887003; doi:10.1002/jcb.29861)
Supplement: Supplementary file 1 — Supporting information. [file JCB-122-335-s001.docx]

Supplemental Table-1. SphK-1 expression in OCLs and S1PR3 expression in OBs in femoral bone sections from 12 months old male and female WT or *MVNP-* mice.

SphK-1 (+) osteoclasts (mm^-1^) S1PR3 (+) osteoblast (mm^-1^)

_____________________________________________________________________________________

Wild Type Male (n=2) 5 ± 1 30 ± 5

Female (n=2) 6 ± 1 28 ± 2

*MVNP* mice Male (n=3) 12 ± 1 30 ± 4

Female (n=2) 15 ± 1 27 ± 3

**_______________________________________________________________**______________________Immunostaining of femoral bone sections for SphK-1 and S1PR3 expression was done as described in Materials and Methods. Positive cells were counted in 3 randomly selected squares per bone section. Results are expressed as the Mean ± SEM. The number of SphK1 (+) OCLS or S1PR3 (+) OBs in bone sections from male vs female mice were not significantly different for each genotype using a two-tailed Welch’s t-test. SphK-1, sphingosine kinase-1; S1PR3, phingosine-1-phosphate receptor 3; WT, wild type; MVNP, measles virus nucleocapsid protein.
